# Integration of Artificial Intelligence and Microfluidics for Drug Delivery Applications

**DOI:** 10.3390/mi17091041

**Published:** 2026-08-31

**Authors:** Shiqi Tang, Xunli Zhang

**Affiliations:** School of Engineering, University of Southampton, Southampton SO17 1BJ, UK

**Keywords:** artificial intelligence (AI), microfluidics, drug delivery systems (DDSs), machine learning (ML), deep learning (DL), organ-on-chip (OoC), nanomedicine

## Abstract

The development of drug delivery systems (DDSs) is moving from empirical, trial-and-error formulation toward data-driven, artificial intelligence (AI)-guided, and automated workflows. Microfluidics provides precise control of microscale fluids, enabling high-throughput production of relatively homogeneous drug carriers such as liposomes, lipid nanoparticles (LNPs), and polymeric micelles. However, the high-dimensional parameter space of microfluidic reactors often exceeds the capacity of manual optimization. This review examines the integration of AI, particularly machine learning (ML), deep learning (DL), and Bayesian optimization, into microfluidic platforms for accelerating DDS design, optimizing critical quality attributes (CQAs), and supporting self-driving laboratory workflows. We discuss the technical foundations of AI-enabled microfluidics, applications in nanocarrier synthesis and phenotypic screening, and the evolving regulatory landscape for AI-assisted pharmaceutical development.

## 1. Introduction

The field of drug delivery systems (DDSs) is shifting from conventional administration strategies toward highly targeted and controlled-release platforms [1,2,3,4]. Traditional macroscale, batch-based synthesis techniques often suffer from limited precision, poor reproducibility, scalability constraints, and variable nanoparticle uniformity. By contrast, microfluidic technology enables precise manipulation of fluids at microliter-to-picoliter scales, creating controlled microenvironments, improving product reproducibility, and supporting tunable material properties for personalized therapies [5,6]. To frame these challenges, it is also useful to consider clinical and preclinical benchmarks in the nanomedicine landscape. For instance, FDA-approved formulations such as Doxil (liposomal doxorubicin) and Onpattro (patisiran, the first approved lipid nanoparticle siRNA formulation) have demonstrated the clinical validity of lipid-based drug delivery. However, the formulation of these multi-component carriers traditionally relies on macroscopic batch mixing, where uncontrolled local concentration gradients lead to batch-to-batch variation, low encapsulation efficiency, and heterogeneous size distributions that compromise in vivo biodistribution [1,3]. Microfluidic technologies directly overcome these constraints by operating at the microscale, where fluidic interfaces are strictly defined [5,6].

Of particular relevance to DDS synthesis, microfluidic systems enable precise control over fluidic parameters, enhancing reaction kinetics, improving drug encapsulation efficiency, and addressing key limitations associated with conventional synthesis approaches [5,7,8]. Moreover, microfluidics-based mechanical delivery strategies, such as constriction- and shear-induced membrane permeabilization, enable high-throughput intracellular cargo delivery with improved transfection efficiency and reduced cellular damage compared with many traditional physical methods [6,9,10].

Despite these advantages, the design and optimization of custom microfluidic platforms remain complex because device geometry, fluid dynamics, formulation composition, and biological response must be controlled simultaneously. Artificial intelligence (AI) can support this process by enabling automated device design, optimization of experimental conditions, and real-time adaptive control [11,12]. AI-driven microfluidic systems can integrate biosensors, machine learning algorithms, and predictive modeling tools to improve drug targeting, personalize dosing strategies, and support more reproducible therapeutic outcomes [5,6].

Furthermore, AI-integrated wearable and implantable microfluidic systems are advancing the development of closed-loop digital therapeutics [13], enabling real-time monitoring and responsive drug release based on patient-specific physiological data [14]. Such systems represent a paradigm shift from static drug administration toward intelligent, autonomous therapeutic platforms [15,16].

Collectively, the convergence of microfluidics and AI is accelerating drug discovery and development workflows, strengthening precision-medicine strategies, and enabling next-generation DDSs that are more efficient, adaptable, and patient-specific than many conventional approaches [9,17]. With appropriate validation, these platforms could become important infrastructure for adaptive and personalized pharmaceutical care.

### 1.1. The Challenge of Drug Delivery Systems (DDSs)

Modern pharmacology has evolved beyond conventional small-molecule drugs toward complex, multi-component drug delivery systems (DDSs) such as lipid nanoparticles (LNPs), liposomes, and other polymer-based nanocarriers [18,19]. These systems are engineered to enhance therapeutic efficacy, enable targeted delivery, and reduce systemic toxicity. Lipid-based nanoparticles, in particular, have demonstrated significant potential for drug and gene delivery, including nucleic acids such as mRNA, and are now central to advanced therapeutic strategies [20,21]. However, despite their promise, industrial development and clinical translation remain challenging due to physicochemical instability, limited reproducibility and scalability, and the need to meet stringent regulatory requirements.

#### 1.1.1. Complexity of Multi-Component Architectures

A defining challenge of DDS development is the high-dimensional design space created by multi-component architectures. For example, lipid-based systems typically include multiple lipid species (e.g., structural lipids, cholesterol, PEGylated lipids), each contributing to physicochemical properties such as size, stability, and cargo interaction [21]. Furthermore, the interaction between lipid composition, processing parameters, and therapeutic payload determines key critical quality attributes (CQAs), including particle size, structural integrity, and encapsulation performance. Computational modeling has increasingly been employed to understand and predict such interactions, highlighting the structural and functional complexity of these systems [21].

Industrial translation further compounds this complexity. Manufacturing often involves multi-batch processes, where scalability, sterility, reproducibility, structure–function correlations, and large-scale production become difficult to control [22]. Such complexity necessitates structured approaches such as Quality-by-Design (QbD) and advanced process analytical technology (PAT) to systematically link formulation parameters with product performance [19,20].

#### 1.1.2. Limitations of Batch Synthesis and Manual Optimization

Traditional macroscale batch synthesis methods generally suffer from batch-to-batch variability, limited scalability, and insufficient control over mixing and nucleation dynamics. These limitations directly affect nanoparticle uniformity and overall quality control [23]. In this context, microfluidics-based manufacturing has emerged as a promising solution, enabling controlled laminar mixing and improved reproducibility. However, despite its technical advantages, microfluidic production still requires labor-intensive, case-by-case optimization due to limited mechanistic understanding and the scarcity of comprehensive datasets covering diverse lipid compositions [24].

Specifically, manual optimization strategies, often based on sequential parameter variation, fail to capture the multidimensional interactions among flow rates, composition, and concentration variables. ML approaches have demonstrated a superior ability to predict liposome formation, size and the key CQAs, highlighting the limitations of conventional optimization methods in navigating such complex formulation spaces [17,24,25]. Furthermore, the transition from batch to continuous production requires the integration of advanced manufacturing technologies and analytical tools to ensure consistent quality and regulatory compliance [20,24].

#### 1.1.3. The Data Gap and Translational Barriers

One of the major barriers to predictive formulation design is the limited availability of comprehensive, high-quality datasets. Microfluidics-based liposome production, for example, suffers from insufficient data diversity across lipid systems, which hinders the development of generalizable predictive models [21,24]. Even with the integration of automation and AI, significant challenges remain, as AI-driven formulation design depends heavily on data quality, dataset scale, and algorithm transparency. Issues related to data integrity, regulatory scrutiny, and ethical considerations, such as algorithmic bias, as well as concerns over reproducibility, transparency, and the need for skilled workforces, further limit immediate large-scale adoption [26,27,28,29,30].

Specifically, this persistent data scarcity stems from three structural issues: (i) the highly proprietary nature of pharmaceutical research, which confines high-quality datasets within corporate silos; (ii) the lack of standardized metadata schemas for microfluidic variables, making it difficult to aggregate data across different experimental platforms; and (iii) publication bias, whereby negative results (e.g., failed synthesis runs that yield highly polydisperse or unstable nanoparticles) are rarely published, depriving machine learning models of critical negative training examples [26,28,29]. To overcome these barriers, the field must establish open-science repositories (similar to the Nanomaterial Registries) with standardized reporting schemas for flow rate ratio (FRR), total flow rate (TFR), organic solvent composition, and concentration parameters [5,6]. Furthermore, federated learning frameworks could enable multi-institutional model training without exposing sensitive raw data, while active learning algorithms could be deployed to systematically generate diverse, high-utility datasets by targeting formulation spaces associated with high uncertainty [26].

### 1.2. The Microfluidic Advantages for Drug Delivery Systems (DDSs)

The emergence of microfluidics has substantially advanced DDS synthesis by shifting key formulation steps from macroscopic batch processing to controlled microscale fluid dynamics. Central to this transition is the ability to manipulate fluids within sub-millimeter channels, where viscous forces dominate over inertial forces and laminar flow typically prevails [5]. This behavior is characterized by the dimensionless Reynolds number (*Re*), defined in Equation (1):

(1) $$Re=\frac{Du\rho }{µ}$$

where *D* is the characteristic diameter of the flow channel, *u* is the fluid velocity, *ρ* is the fluid density, and *μ* is the dynamic viscosity.

While turbulence typically arises at Reynolds numbers of several thousand in pipe flow, microfluidic systems generally operate at low *Re*, often with values of around 1 [5]. This constrained physical environment enables a high degree of control over molecular assembly, offering a level of precision that is difficult to achieve in conventional macroscale reactors such as stirred tanks.

#### 1.2.1. Precision Engineering and Homogeneity

**Figure 1:**
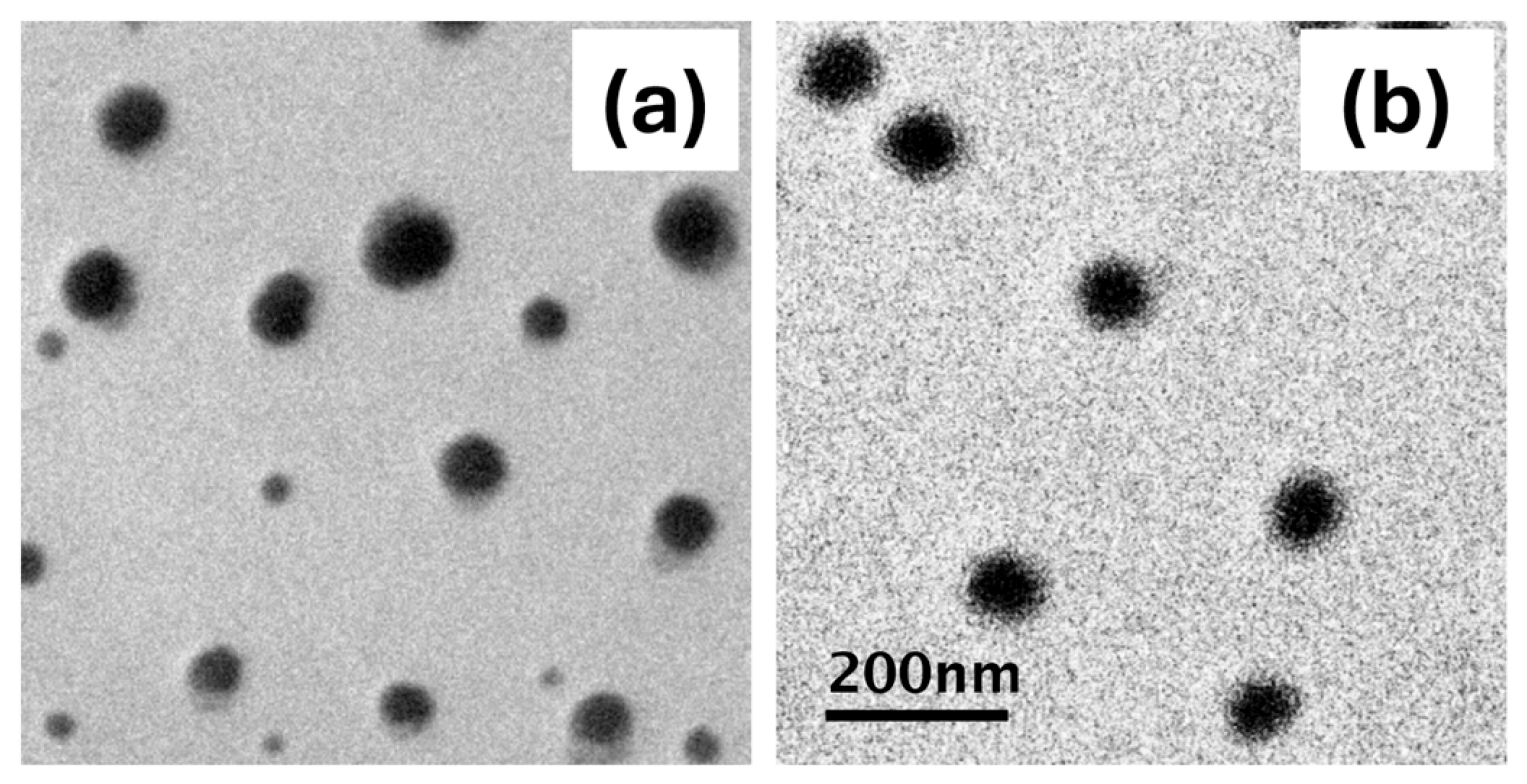
TEM images of polymeric micelle-based drug delivery nanoparticles synthesized from (**a**) batch and (**b**) continuous-flow microfluidic reactors.

One of the most significant advantages of microfluidic reactors is precise control over mixing kinetics. In continuous-flow systems, such as staggered herringbone mixers or flow-focusing geometries, an organic phase containing active compounds mixes with an aqueous phase through rapid diffusion across controlled interfaces [31,32,33]. This controlled environment helps particles experience more uniform chemical conditions during nucleation and growth. Microfluidic synthesis can therefore produce nanocarriers with low polydispersity indices, although achievable PDI depends on formulation chemistry, reactor design, and operating conditions [34]. Figure 1 compares micelle-based drug delivery nanoparticles synthesized using batch and microfluidic reactor systems, illustrating the improved size homogeneity achievable with microfluidic reactors [32,33].

Physically, rapid mixing is paramount because it dictates the final nanoparticle size and homogeneity. When mixing occurs on a timescale faster than the characteristic precipitation/nucleation time, the nucleation phase is completely separated from the subsequent growth phase [5,6]. This separation ensures that all nanoparticles nucleate simultaneously under identical concentration conditions, leading to exceptionally monodisperse size distributions. Because microfluidic channels operate under strictly laminar flow regimes (low Re), mixing in a simple straight channel relies entirely on transverse molecular diffusion, which is slow over macro-scale widths [7]. Therefore, specialized channel geometries are required: hydrodynamic flow focusing narrows the fluid stream to reduce diffusion distances (as diffusion time scales quadratically with distance), while staggered herringbone mixers split, rotate, and fold the fluid layers to exponentially increase the interfacial contact area, thereby achieving rapid mixing within milliseconds even in the absence of turbulence [5,6,7].

#### 1.2.2. High-Throughput Data Generation and Scalability

Unlike batch synthesis, where scale-up often requires re-optimization because surface-area-to-volume ratios and heat-transfer rates change, continuous-flow microfluidics can support scale-out through the parallelization, or numbering-up, of identical chips [35]. The continuous nature of the process also enables high-throughput screening of formulation libraries. By modulating syringe-pump speeds or using automated liquid handlers, a single microfluidic platform can generate hundreds of LNP or micelle formulations in substantially less time than many batch workflows. This capability makes microfluidics a strong partner for AI-driven optimization because it can generate the standardized datasets needed to train ML models [36].

#### 1.2.3. Real-Time Monitoring and Reproducibility

In-line analytical tools further distinguish continuous-flow microfluidic platforms. Dynamic light scattering (DLS), UV-Vis spectroscopy, and small-angle X-ray scattering (SAXS) can be coupled to reactors to monitor nanoparticle size and stability in real time [37,38]. These PAT-compatible measurements enable feedback control, in which synthesis parameters are adjusted to keep CQAs within predefined limits. This capability is particularly relevant for QbD-oriented development and for future self-driving laboratory workflows [39,40,41]. Thus, microfluidics provides the physical control needed for reproducible formulation, while AI provides the computational layer needed to optimize nonlinear design spaces and operate adaptive workflows.

### 1.3. AI-Enabled Transition in Microfluidics-Based Drug Delivery

The integration of AI into microfluidic drug delivery synthesis marks a transition from mainly heuristic experimentation toward more data-driven and automated workflows. While microfluidics addresses important physical limitations of batch processing, continuous-flow reactors generate multidimensional datasets that require computational frameworks capable of navigating complex parameter spaces. In this context, AI enables a progression from passive data analysis to predictive modeling, sequential experimental design, and, in selected cases, autonomous decision-making [42].

#### 1.3.1. Beyond Heuristics: Machine Learning for Global Optimization

Traditional optimization in drug delivery development often relies on the intuition of experienced researchers or on restrictive statistical approaches such as response surface methodology (RSM). However, RSM suffers from severe limitations: (i) it assumes relatively simple quadratic or polynomial relationships, making it incapable of capturing highly non-linear, multi-modal landscapes; (ii) it is highly sensitive to experimental noise; and it fails as the number of design variables increases, requiring an impractical number of runs to map high-dimensional formulation spaces. In contrast, Bayesian optimization (BO) and supervised machine learning can model complex, high-dimensional spaces without assuming a fixed algebraic form. In principle, BO employs a surrogate model, commonly a Gaussian process, to describe the relationship between input parameters (e.g., flow rate and chemical composition) and output quality attributes (e.g., particle size and polydispersity) [36]. Literature examples highlight the power of these methods, allowing researchers to evaluate thousands of virtual formulations in silico before synthesizing them in the lab.

Unlike conventional methods, BO uses an acquisition function to balance the exploration of uncertain regions of the parameter space with the exploitation of known high-performing conditions. Recent studies have shown that AI-guided optimization can identify optimal formulation windows up to an order of magnitude faster than manual tuning, while significantly reducing the consumption of costly reagents such as ionizable lipids or mRNA [35,43].

#### 1.3.2. Deep Learning and the Rise of Physical AI

While standard ML is useful for optimization, deep learning (DL) is increasingly applied to complex pattern-recognition and simulation tasks. Convolutional neural networks (CNNs) are now used for real-time droplet classification and morphology detection, providing a level of granularity in quality control that is difficult to achieve by manual observation alone. Physics-informed neural networks (PINNs) represent another important development because they incorporate governing equations, such as the Navier-Stokes equations, into the training objective. This can allow AI models to predict flow behavior and mixing patterns within microchannels while reducing reliance on computationally expensive computational fluid dynamics (CFD) simulations [44,45].

By embedding governing physical laws into neural network training, physics-informed or physical AI models unify mechanistic understanding with data-driven learning. This integration enhances model robustness, interpretability, and generalizability, ensuring that predicted outcomes remain physically and biologically plausible [46,47,48].

#### 1.3.3. Towards the Self-Driving Lab (SDL)

A major expression of this transition is the self-driving laboratory (SDL), in which AI acts as the decision-making layer within a closed-loop discovery system. In SDL, algorithms design experiments, initiate automated synthesis or testing workflows, collect data through integrated sensors, update predictive models in real time, and select subsequent experimental conditions with limited human intervention. The AGILE platform exemplifies this direction by combining DL with combinatorial chemistry to accelerate the discovery of ionizable lipids for mRNA delivery, which can be optimized by neural networks, a deep-learning strategy for ionizable lipid design [34,43,49].

By reducing human bias and minimizing inefficient trial-and-error strategies, SDLs accelerate nanocarrier discovery while improving reproducibility, traceability, and standardization of documentation. This transition from laboratory automation to true scientific autonomy represents a critical step toward meeting the demands of personalized medicine, where rapid therapeutic optimization may directly impact patient outcomes [39,50,51].

### 1.4. Scope and Objectives of the Review

This review aims to provide a comprehensive overview of the convergence between microfluidic and artificial intelligence technologies in the modern pharmaceutical landscape. It examines the transition from manual, bench-top synthesis to the sophisticated, autonomous paradigms that define contemporary drug delivery research.

Special emphasis is placed on the technical foundations of AI-integrated microreactors, including specific architectures such as hydrodynamic focusing and droplet-based systems, as well as the diverse AI toolsets, ranging from supervised learning to Bayesian optimization, that govern these platforms. The review also explores the application of these technologies in the precision engineering of delivery vehicles, such as lipid nanoparticles (LNPs) and solid lipid nanoparticles (SLNs), and their subsequent evaluation in high-content phenotypic screening models, including Organ-on-Chip (OoC) systems.

Furthermore, it discusses the mechanics of the closed-loop Self-Driving Lab and the role of advanced modeling techniques, such as PINNs and digital twins. Finally, the review outlines evolving regulatory considerations proposed by the FDA, providing a critical analysis of the current challenges and future perspectives that will shape the next generation of AI-driven development of drug delivery systems.

While several reviews separately address AI in drug discovery or general microfluidic engineering, this review occupies a unique and highly focused niche. Specifically, it examines the direct, closed-loop integration of physical microfluidic hardware with machine intelligence for DDS synthesis and phenotypic screening. Unlike existing literature, this review (i) systematically maps how physical microfluidic parameters (e.g., fluid shear, chaotic advection, and droplet compartment dynamics) translate into digital descriptors used by machine-learning models; (ii) examines how high-content phenotypic screening, such as 3D tumour-on-chip models, can provide real-time biological feedback to drive AI-based DDS formulation optimization; and (iii) analyzes these advances through the lens of the FDA’s newly proposed 2025 risk-based credibility assessment framework for AI in drug development. This review thus bridges the physical–digital divide, offering a roadmap towards true laboratory autonomy (i.e., self-driving laboratories) in precision nanomedicine.

## 2. Technical Foundations of Microfluidics and AI

This section introduces the microfluidic architectures most relevant to nanocarrier production and explains why their controlled, data-rich operating conditions are well suited to AI-guided optimization.

### 2.1. Microfluidic Architectures for DDSs

Rather than repeating the general advantages of microfluidics, the following subsections focus on how specific architectures create different opportunities and constraints for DDS synthesis.

For oncology and other targeted-delivery applications, particle size, surface chemistry, and release behavior strongly influence pharmacokinetics and therapeutic performance [34,52]. Microfluidic design can support passive targeting through particle geometry, active targeting through surface functionalization, and stimuli-responsive release through pH- or thermosensitive polymer incorporation. The remaining challenge is that these architecture–property relationships are nonlinear and formulation-specific, which motivates the AI methods discussed below.

#### 2.1.1. Microfluidic Hydrodynamic Focusing (MHF)

**Figure 2:**
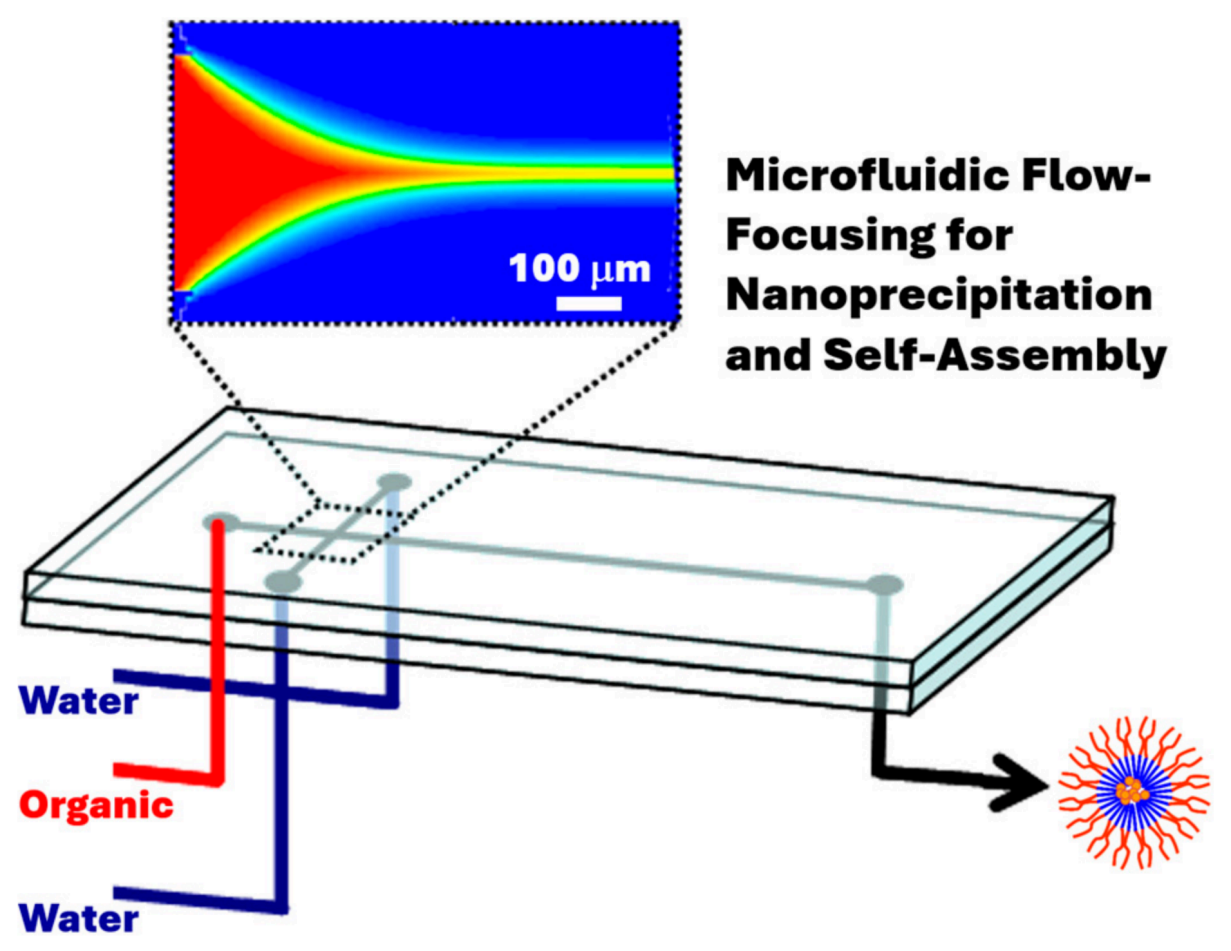
Microfluidic hydrodynamic focusing (MHF) reactor for nanoprecipitation and self-assembly of polymeric micelle-based drug delivery systems. Insert: CFD simulation showing the diffusive mixing process along the main flow channel, where the green area represents the mixed region of water (blue) and organic solution (red).

Microfluidic hydrodynamic focusing (MHF) serves as a cornerstone architecture for the bottom-up synthesis of drug delivery vehicles. In this configuration, a central lipid-containing solvent stream is sandwiched between two flanking aqueous buffer streams at a microfluidic junction. The resulting laminar flow establishes a highly controlled interface where molecular self-assembly is governed exclusively by transverse diffusion. As the aqueous buffer migrates into the central stream, the local solvent concentration decreases, triggering the supersaturation and subsequent nucleation of lipid components into nanoparticles. Figure 2 illustrates an exemplary MHF reactor system for the synthesis of polymeric micelle-based nanostructured DDSs [32,33].

The primary advantage of MHF is that the mixing time can be tuned by adjusting the flow-rate ratio (FRR) between the aqueous and organic phases. By narrowing the width of the focused central stream, diffusion distances are reduced, enabling rapid mixing that can outpace particle-growth kinetics. The time (*t*) required for a molecule to diffuse across a distance (*L*) is governed by Fickian diffusion [53], as simplified in Equation (2), where *D* is the diffusion coefficient.

(2) L=2D·t12

This kinetic control is essential for producing particles with narrow size distributions and high batch-to-batch consistency [33]. Furthermore, because MHF maintains the reaction zone away from the channel walls, it significantly reduces the risk of reactor fouling or clogging, which is common in traditional bulk synthesis. This stability makes MHF particularly compatible with AI-driven feedback loops, as the predictable relationship between fluidic parameters and particle size provides a reliable basis for predictive modeling [36,54].

#### 2.1.2. Chaotic Mixing

Chaotic mixing architectures address the limitations of diffusion-dominated mixing in laminar flow by using engineered microstructures to induce chaotic advection. Structures such as staggered herringbone mixers (SHMs) [55,56,57] and Tesla arrays [58,59,60,61] create transverse flow components that repeatedly stretch and fold fluid interfaces. This process increases the surface area available for diffusion and can reduce mixing times from seconds to milliseconds.

Specifically, SHMs are exceptionally prevalent in the synthesis of complex multicomponent nanocarriers, such as ionizable lipid nanoparticles (LNPs) for mRNA delivery, for two thermodynamic and fluidic reasons: (i) mRNA is highly prone to degradation and requires immediate, uniform electrostatic complexation with ionizable lipids to form stable, protective cores; and (ii) the staggered herringbone grooves generate counter-rotating helical flows (vortices) that stretch and fold the organic and aqueous streams [55,56,57]. Mathematically, chaotic advection causes the characteristic diffusion length to decrease exponentially with increasing residence time, and hence with downstream distance under steady-flow conditions, while the characteristic mixing behavior is governed by the Péclet number, *Pe*, which describes the relative contributions of advective transport and molecular diffusion.

(3) Pe=u·L/D

where *u* is the mean fluid velocity, *L* is the characteristic transverse dimension of the channel (e.g., its width or hydraulic diameter), and *D* is the molecular diffusion coefficient of the diffusing species. This rapid stretching and folding reduces the diffusion length scale to the micrometer range, enabling near-complete homogenization within milliseconds. Under these rapid and spatially uniform mixing conditions, the self-assembly of the LNP components (including the ionizable lipid, cholesterol, helper lipid, and PEG-lipid) with mRNA occurs concurrently and reproducibly throughout the fluid volume, promoting efficient and consistent encapsulation of the nucleic-acid cargo [55,56,57].

The use of SHMs is particularly prevalent in the synthesis of complex multicomponent nanocarriers, such as ionizable lipid nanoparticles for mRNA delivery. By achieving rapid homogenization, these mixers ensure that the self-assembly of lipids and nucleic acids occurs under uniform conditions, which is paramount for maximizing encapsulation efficiency [55,56,57]. Tesla arrays and other passive micromixers further enhance this by providing high-throughput capabilities without the need for moving parts, thereby increasing system robustness [58,59,60,61].

From an AI perspective, chaotic mixing presents a more complex computational challenge than MHF due to the intricate fluid–structure interactions involved. Consequently, recent efforts have focused on using DL to optimize these mixer geometries, ensuring that advection-enhanced homogenization is achieved with minimal pressure drop and shear stress [34].

#### 2.1.3. Droplet-Based Microfluidics

**Figure 3:**
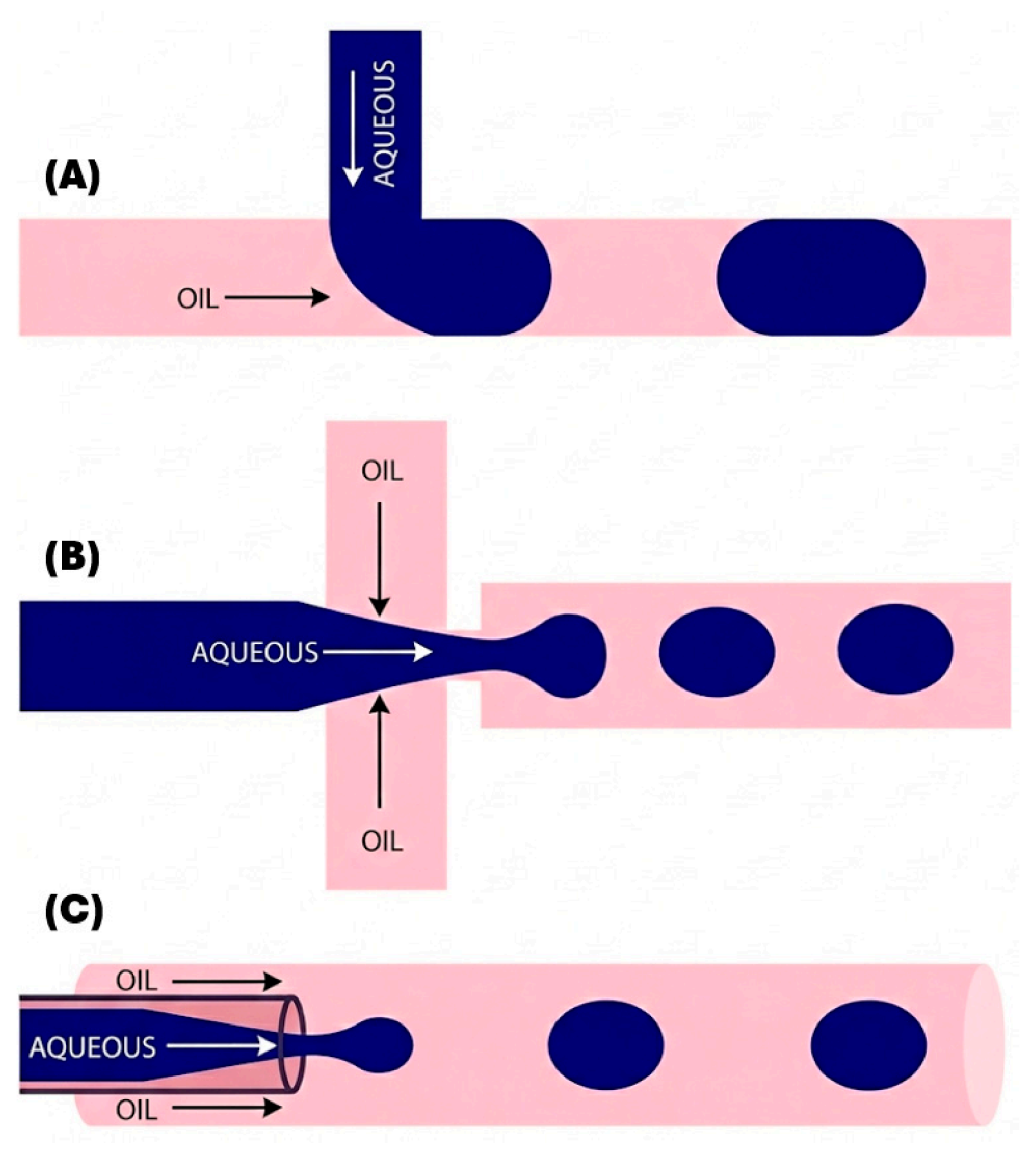
Different microfluidic configurations for the formation of droplets with (**A**) T-junction, (**B**) hydrodynamic flow-focusing, and (**C**) co-flowing microchannel geometries.

Droplet-based microfluidic systems use immiscible fluids, typically an aqueous phase and an oil phase, to generate discrete, monodisperse microdroplets that serve as picoliter-scale reaction vessels [62]. Within droplets, internal circulation promotes rapid mixing and enhanced mass transfer [63]. Figure 3 illustrates common modes of droplet formation in microfluidic channels [64].

Droplet compartmentalization is especially useful for drug delivery research because each droplet can function as an isolated reaction or screening unit. This supports parallel exploration of formulation variables, reduces cross-contamination, and enables high-throughput screening of drug candidates or carrier-cell interactions [62,63,65].

Numerous studies demonstrate the utility of droplet-based continuous flow microreactors in the literature [62,63,64,65]. For instance, droplet microfluidics has been successfully used to synthesize monodisperse PLGA microspheres with highly consistent drug loading and controlled-release profiles, bypassing the large size variation in traditional emulsion techniques. In screening applications, droplet platforms have been used to generate highly monodisperse water-in-oil-in-water (W/O/W) double emulsions to encapsulate combination drug cocktails, enabling the high-throughput screening of synergistic chemotherapy drug ratios on breast cancer cell lines. Furthermore, single-cell encapsulation within microdroplets has enabled researchers to screen the cellular uptake and cytotoxicity of diverse LNP libraries at unprecedented throughput, capturing cell-to-cell variability that is obscured in bulk assays.

Because each droplet can also generate imaging or sensor data, droplet microfluidics pairs naturally with AI-enabled analysis. CNNs, deformable detection transformer (DETR), and related models can classify droplet morphology, encapsulation events, or biological responses in real time, supporting automated drug-testing platforms [29,65,66,67].

### 2.2. AI Toolsets for Microfluidic DDS Optimization

In microfluidic drug delivery, AI can function as the computational engine that interprets high-frequency, multidimensional data streams generated by continuous-flow microreactors [68]. Microfluidic synthesis platforms enable precise control over formulation parameters, including total flow rate, aqueous-to-organic flow-rate ratio, and precursor concentration. However, the relationships between these inputs and resulting CQAs, such as particle size, polydispersity index, stability, and encapsulation efficiency, are often nonlinear and difficult to resolve using conventional statistical approaches. Supervised ML models, including support vector machines (SVMs), random forests, and artificial neural networks (ANNs), have shown strong predictive capability in capturing these relationships and predicting nanoparticle size, dispersity, stability, and loading efficiency from synthesis parameters [24,69,70].

In practice, these models can move microfluidic optimization from retrospective analysis toward prescriptive control: they can recommend synthesis conditions that target user-defined CQAs while reducing material consumption, which is especially valuable for expensive therapeutic payloads or lipid libraries [24,70]. Hybrid modeling approaches are also emerging, combining ensemble learning, kernel methods, DNNs, and interpretability tools such as SHAP to maintain predictive performance while improving mechanistic insight [24,68,70].

#### 2.2.1. Supervised Learning

Supervised learning involves training algorithms on labeled historical datasets in which both input parameters and output results are known. In DDS applications, this paradigm is primarily used for regression and classification tasks, such as predicting particle size, zeta potential, or drug encapsulation efficiency from formulation variables [71,72]. Common architectures include random forests, support vector regression (SVR), and gradient-boosted trees, which can identify factors such as lipid-to-drug ratio or aqueous-phase pH that strongly influence product stability [73,74].

Beyond simple prediction, supervised learning models can also serve as the foundation for digital twins of microfluidic reactors. By training on thousands of previous synthesis runs, these models can provide real-time estimates of CQAs when physical sensors might be delayed or unavailable. This predictive capability is vital for maintaining batch-to-batch consistency in the production of complex biologics [75,76].

However, the efficacy of supervised learning is inherently limited by the quality and diversity of the training data. This limitation motivates active learning, in which the model identifies the next most informative experiments needed to improve predictive accuracy [77].

To provide a clear and realistic assessment of AI’s role, we must distinguish between AI technologies that have been experimentally demonstrated in physical microfluidic setups, and those that currently remain conceptual or predictive (Table 1):

- Experimentally Demonstrated AI: These tools are directly integrated with physical hardware. For example, Bayesian Optimization (BO) has been coupled with syringe pumps and dynamic light scattering (DLS) to perform closed-loop, autonomous optimization of lipid nanoparticles, identifying optimal flow rate ratios (FRRs) and compositions in real time. Similarly, supervised Artificial Neural Networks (ANNs) and Random Forests (RFs) have been trained on real laboratory runs to predict liposome size and encapsulation efficiency, and Convolutional Neural Networks (CNNs) are actively used in-line for real-time droplet size classification and sorting.
- Conceptual and Predictive AI: These tools are currently confined to computational simulations and predictive modeling. For example, Physics-Informed Neural Networks (PINNs) incorporate Navier–Stokes equations to simulate microfluidic mixing and optimize channel geometries without requiring physical fabrication, but they are not yet integrated into real-time physical feedback control loops. Similarly, Digital Twins of full pharmaceutical manufacturing plants that predict long-term equipment drift or product degradation remain conceptual frameworks that are not yet deployed in physical GMP production due to a lack of certified sensor integration and regulatory guidelines.

#### 2.2.2. Unsupervised Learning

Unsupervised learning is employed to discover hidden patterns and structures within unlabeled data, making it an indispensable tool for exploratory research in microfluidics. In droplet-based systems, for example, unsupervised algorithms like K-means clustering or principal component analysis (PCA) are used to identify distinct subpopulations of droplets or cells based on high-dimensional phenotypic data [83,84]. This is particularly useful in identifying outlier events in nanoparticle morphology or detecting subtle changes in droplet stability that might be missed by human observers or predefined threshold-based systems [85].

Furthermore, unsupervised learning facilitates dimensionality reduction in complex datasets, allowing researchers to visualize the landscape of a formulation space. By identifying which parameters naturally cluster together, these models can suggest novel combinations of lipids or polymers that have not been previously explored. Recently, self-supervised learning as a subset of unsupervised learning is increasingly used to pre-train models on vast amounts of raw video data from microfluidic channels, enabling more robust downstream tasks like automated anomaly detection or real-time process monitoring without the need for exhaustive manual labeling [86].

#### 2.2.3. Reinforcement Learning (RL)

Reinforcement Learning (RL) represents a shift toward active control, where an autonomous agent learns to navigate a process through trial and error to maximize a defined reward signal. In microfluidic synthesis, RL agents are increasingly deployed to manage the real-time dynamics of syringe pumps and pressure controllers [87,88]. By receiving constant feedback from in-line sensors, the agent can adjust flow rate ratios or total flow rates to compensate for pressure fluctuations or minor clogs, ensuring that the synthesis remains within the optimal zone for particle formation [87,89].

The value of RL lies in its ability to handle non-stationary environments where the optimal strategy may change over time because of reactor fouling, pressure drift, or reagent degradation. Advanced deep RL (DRL) architectures, which combine neural networks with reinforcement learning principles, can learn complex control policies that may outperform traditional proportional–integral–derivative (PID) controllers in selected dynamic control problems [90,91]. This makes RL a plausible component of self-driving laboratory systems, allowing autonomous recovery from process disturbances and helping maintain product quality with reduced human intervention [39].

#### 2.2.4. Deep Learning (DL)

DL uses multilayered neural networks to extract high-level features from complex, high-dimensional data. In microfluidic drug delivery, CNNs have become effective tools for image-based tasks, including real-time classification of droplet shapes and detection of nanoparticle aggregation within channels [83]. These models can process large image streams and provide automated vision systems for synthesis-quality monitoring. Recurrent neural networks (RNNs) and Transformer-based architectures are also applied to sequential industrial data, such as temperature or pressure time series, to model temporal dependencies and predict future process states [92,93]. These approaches support anomaly detection and predictive monitoring, allowing potential system failures to be identified earlier [94].

In this setting, PINNs are most valuable as design and simulation tools: by incorporating fluid-dynamic constraints, they can estimate how geometry or viscosity affects mixing efficiency without requiring full-scale CFD for every candidate design [44,45,46,47,48].

#### 2.2.5. Bayesian Optimization (BO)

BO is widely recognized as a powerful strategy for global optimization of expensive experimental systems, making it particularly suitable for drug formulation and drug delivery research where each experiment may require significant time, materials, or analytical effort [95]. BO operates by constructing a probabilistic surrogate model of the experimental design space, most commonly a Gaussian Process (GP), which approximates the relationship between formulation variables and experimental outcomes [96]. An acquisition function is then used to sequentially select the next experiment, balancing exploration of uncertain regions of the design space with exploitation of regions predicted to yield high performance. This sequential decision-making framework enables efficient identification of optimal formulation conditions while substantially reducing the number of required experiments compared with traditional approaches such as grid search or random experimentation. Consequently, BO has become an increasingly important tool for accelerating formulation development and reducing experimental costs in pharmaceutical research [96,97].

A key advantage of BO in microfluidic experimentation is its ability to explicitly model noise and uncertainty in experimental measurements. It provides not only predictions of experimental outcomes but also quantitative uncertainty estimates, which is particularly valuable for microfluidic systems where variability may arise from fluctuations in flow conditions, mixing dynamics, or measurement noise [81]. Unlike deterministic optimization methods, BO incorporates this uncertainty into its decision-making process, enabling more robust exploration of the experimental design space [95].

In drug delivery research, BO and other machine-learning-driven optimization strategies have been increasingly integrated with microfluidic platforms for lipid nanoparticle (LNP) formulation screening, where multiple formulation variables, such as lipid composition, flow rate ratios, and solvent conditions, must be tuned simultaneously. Microfluidic technologies enable the rapid generation of diverse nanoparticle libraries and facilitate high-throughput exploration of formulation parameters, while data-driven optimization methods accelerate the identification of high-performance formulations with improved transfection efficiency or delivery performance [98,99].

## 3. AI-Enhanced Synthesis of Nano-Drug Delivery Systems

The primary goal in synthesizing nano-DDSs is to control CQAs, particularly nanoparticle size, polydispersity index (PDI), and encapsulation efficiency (EE). While microfluidic systems provide the physical means for high-resolution mixing, AI and ML models are changing how these processes are optimized. Predictive models, often based on ANNs, can function as in silico screening tools, estimating how variables such as polymer concentration and flow rate influence nanoparticle size before reagents are consumed [27,28].

In these complex self-assembly systems, experimental noise and physical variability (e.g., micro-fluctuations in syringe pump pressure, local temperature drifts, or minor raw material impurities) represent significant optimization challenges [27]. AI models handle this high variability through robust optimization techniques. For example, BO utilizes Gaussian Process (GP) surrogate models that natively incorporate a noise hyperparameter (representing experimental variance). This allows the algorithm to distinguish between a genuine performance peak and a random spike caused by noise, preventing the system from selecting unstable, high-variance operating points. Additionally, active learning frameworks incorporate uncertainty quantification (using acquisition functions like Expected Improvement or Upper Confidence Bound) to guide exploration, deliberately sampling regions where experimental noise is high to build a more resilient global model. Ensemble methods, such as Random Forests, also buffer against local experimental errors by averaging predictions across hundreds of independent decision trees [11,28].

Beyond predictive modeling, AI enables high-frequency, real-time monitoring and control of microfluidic processes. CNNs have been integrated into microfluidic imaging systems for automated droplet detection, classification, and feedback control, allowing droplet-formation dynamics to be monitored at millisecond timescales [65,100]. Such systems can rapidly detect deviations in droplet size, frequency, or morphology and respond to disturbances in fluid flow, such as pressure fluctuations or minor channel blockages, thereby improving process stability and reproducibility.

Together, real-time imaging, predictive modeling, and XAI provide a practical route from black-box optimization toward interpretable microfluidic process control. These approaches enhance model transparency and facilitate the transition from purely predictive black-box models toward more interpretable, data-driven frameworks for microfluidic system design and drug delivery optimization [101,102].

### 3.1. Lipid-Based Nanoparticles (LNPs) and Liposomes

Microfluidic technologies enable the bottom-up synthesis of lipid-based nanoparticles, which have become the leading delivery platform for mRNA therapeutics [35,54,98,99]. In these systems, nanoparticles are formed through the rapid mixing of lipid-containing organic phases with aqueous buffers, resulting in the self-assembly of particles composed of ionizable lipids, cholesterol, phospholipids, and PEGylated lipids. The relative composition of these components strongly influences critical properties such as particle size, polydispersity, encapsulation efficiency, and biological activity. Because these processes involve complex interactions among formulation variables and flow conditions, AI and ML approaches are increasingly used to guide the optimization of microfluidic nanoparticle synthesis [54,68,78,98].

ANNs, in particular, have demonstrated strong predictive capabilities for nanoparticle formation in microfluidic systems [70,102,103]. Studies have shown that ANN models trained on experimental datasets can accurately predict liposome or nanoparticle size and polydispersity based on key processing parameters. This indicates that ML models can effectively map the multidimensional synthesis landscape and enable rapid identification of optimal operating conditions [78,104].

**Figure 4:**
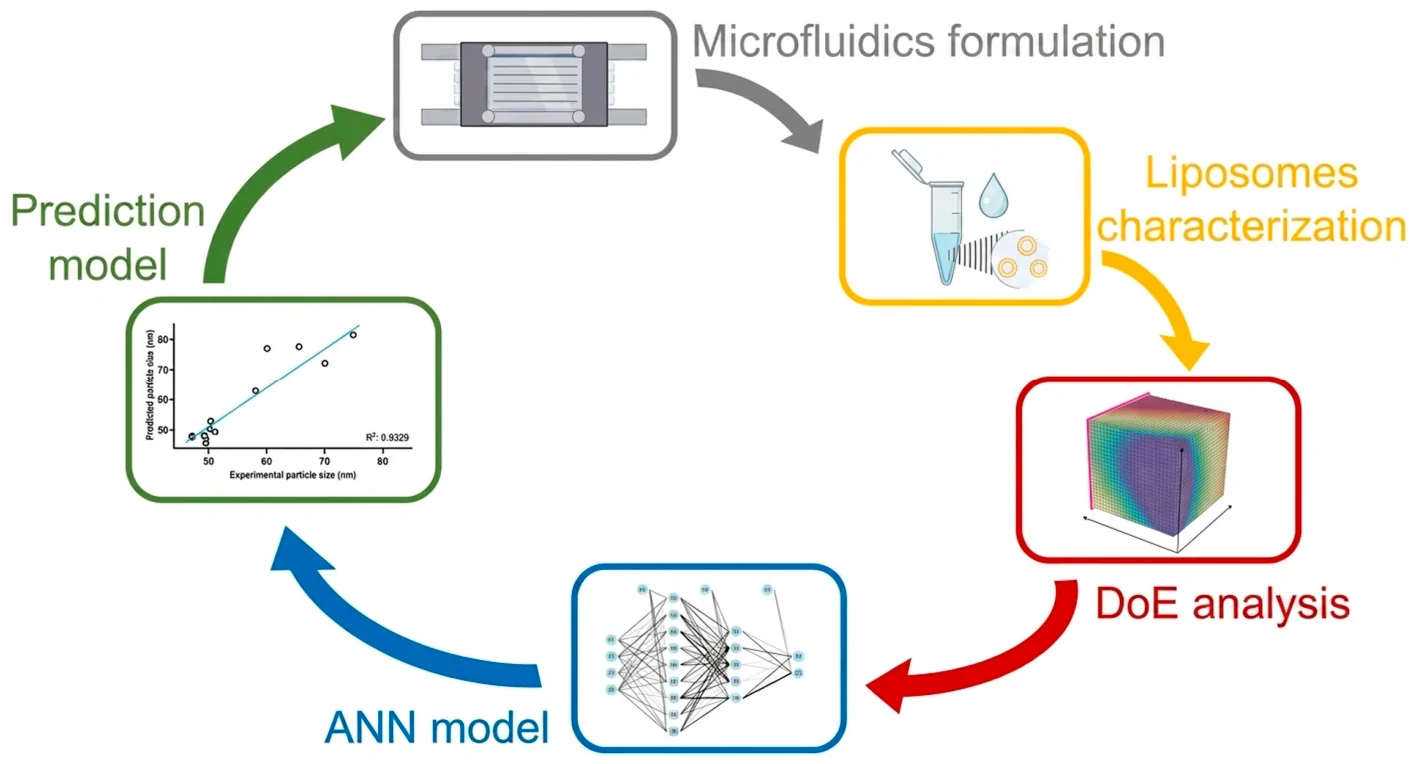
Schematic of the ML-guided liposome optimization workflow, integrating a microfluidic chip with supervised learning models [78] (reproduced with permission; copyright 2022 American Chemical Society).

For example, in the study by Rebollo et al. [78], a combined strategy involving Design of Experiment (DoE) and ML was employed to optimize the microfluidic synthesis of liposomes (Figure 4). Key process parameters, including flow rate ratio (FRR) and total flow rate (TFR), were systematically varied using a microfluidic hydrodynamic focusing (MHF) platform. A suite of ML models, including ANNs, Random Forest (RF), and Support Vector Regression (SVR), were then trained on the resulting experimental dataset. The study demonstrated that the ANN model achieved a cross-validated R^2^ exceeding 0.97 for liposome size prediction, enabling accurate mapping of the high-dimensional parameter space and rapid identification of synthesis conditions that yielded liposomes with sub-200 nm diameters and a PDI below 0.12 [78].

This integrated DoE-ML workflow significantly reduced the number of physical experiments required for process optimization, illustrating how ML-guided microfluidic synthesis can overcome the inherent inefficiencies of traditional trial-and-error formulation development.

The transition toward AI-guided nanoparticle development has also emphasized the importance of model interpretability to better understand the underlying physicochemical relationships governing nanoparticle formation. By integrating XAI tools, researchers can identify which formulation parameters, such as buffer pH, lipid composition, or cholesterol fraction, most strongly influence outputs including particle size, zeta potential, or polydispersity index [102]. These interpretability tools enable mechanistic insight into complex datasets and improve confidence in AI-driven decision-making. Moreover, transparent modeling frameworks are particularly valuable in pharmaceutical development, where regulatory approval requires a clear understanding of the factors controlling product quality and reproducibility [70].

### 3.2. Polymeric Nanoparticles and Hydrogels

Microfluidic platforms provide precise regulation over the fabrication of polymeric nanoparticles such as micelles and polymersomes [32,56,105,106], which are critical for controlled-release applications and tissue engineering. AI is used to optimize these processes by modeling the complex effects of polymer concentration and hydrodynamic parameters on the degradation rate and drug release kinetics [107]. High-throughput droplet microfluidics, when coupled with DL, can screen thousands of antisolvent-crystallization conditions for active pharmaceutical ingredients, ensuring that the resulting polymer–drug composite has the desired crystalline structure and bioavailability [108].

Reviewing these applications in more detail, machine learning has proven highly effective at modeling functional performances, such as hydrogel swelling behavior and PLGA drug loading/release profiles. For instance, ANNs and Support Vector Machines have been trained on cross-linking density, monomer composition, and environmental pH/temperature variables to accurately predict the swelling states of responsive hydrogels, enabling in silico tuning before physical fabrication. Similarly, in the context of PLGA microspheres, Random Forest models trained on curated literature datasets (incorporating polymer molecular weight, LA/GA ratios, solvent types like dimethyl carbonate or ethyl acetate, and FRR) have achieved R^2^ values of 0.96 for encapsulation efficiency and 0.93 for drug loading [80]. These predictive models can simulate how different PLGA formulations release their therapeutic payload over weeks, mapping how polymer chain length and ester-end capping govern hydrolysis and subsequent drug diffusion. This allows researchers to rationally design sustained-release depots without tedious, month-long empirical trials.

In addition to traditional nanoparticle systems, AI-enabled microfluidic platforms are increasingly being used to design and fabricate smart hydrogels that respond to environmental stimuli such as temperature, pH, or ionic strength. ML models can predict key hydrogel properties, including swelling behavior, mechanical strength, and responsiveness, by analyzing synthesis parameters such as cross-linking density, monomer composition, and processing conditions during the fabrication stage. These predictive frameworks enable researchers to establish relationships between material composition and functional performance, facilitating the rational design of advanced hydrogel drug delivery systems. For example, ANNs and other ML algorithms have been successfully used to predict swelling states of responsive hydrogels from synthesis parameters, enabling targeted property tuning prior to experimental validation [109].

More broadly, ML-driven approaches are transforming hydrogel development by enabling data-driven optimization of polymer networks and injectable biomaterials. By integrating experimental data with predictive models, researchers can identify optimal formulations for injectable or printable hydrogel systems used in drug delivery, tissue engineering, and regenerative medicine. These materials can be engineered to form patient-specific drug depots that are either injectable or compatible with 3D bioprinting technologies, thereby enabling controlled therapeutic release and customized treatment strategies [110,111]. Furthermore, ML-driven feedback loops facilitate rapid exploration of complex design spaces and predictive screening of candidate formulations, reducing the number of required experiments and accelerating optimization cycles in hydrogel-based drug delivery research [112,113].

**Figure 5:**
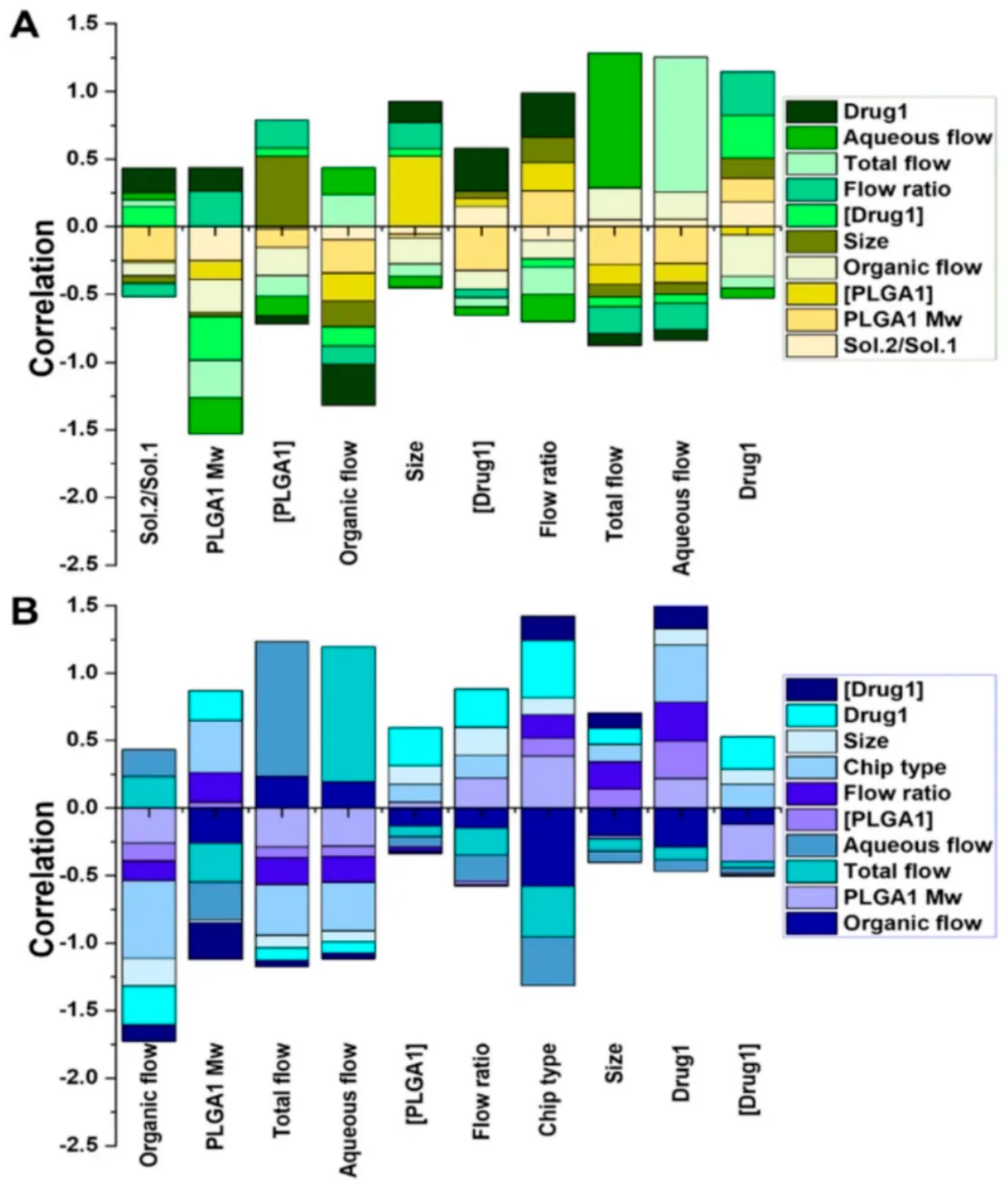
Correlation between selected features for modeling of (**A**) encapsulation efficiency (EE) and (**B**) drug loading (DL) for PLGA nanoparticle formulation [80] (open-access CC-BY; no permission required).

A particularly relevant exemplar study is the work of Hanari et al. [80], which developed ML models to predict critical quality attributes of PLGA-based polymeric nanoparticles synthesized using microfluidic platforms. The authors assembled a curated dataset comprising over 300 PLGA nanoparticle formulations extracted from the literature, encoding 25 physicochemical and process-related features associated with microfluidic preparation conditions, including polymer molecular weight, LA/GA ratio, solvent type, FRR, and the presence of surface-modifying agents such as PEG. Multiple ML algorithms were benchmarked, with the Random Forest model emerging as the top performer, achieving R^2^ values of 0.96 and 0.93 for encapsulation efficiency (EE) and drug loading (DL) prediction, respectively (Figure 5).

Notably, the study found that EE and DL behave as largely independent predictors, providing complementary and non-redundant insights into nanoparticle formulation performance. SHAP-based feature importance analysis further identified polymer molecular weight, LA/GA ratio, and FRR as the most influential parameters governing EE, reinforcing their mechanistic relevance to the nanoprecipitation process. Collectively, this work demonstrates that data-driven ML frameworks trained on aggregated microfluidic formulation datasets can serve as powerful in silico design tools for accelerating PLGA nanoparticle development, substantially reducing the experimental burden associated with traditional empirical screening.

### 3.3. Solid Lipid Nanoparticles (SLNs)

Solid lipid nanoparticles (SLNs) are a promising alternative to liquid-phase drug carriers, offering improved protection and stabilization of lipophilic drugs [54]. However, SLNs are prone to polymorphic transitions and unexpected gelation during the cooling phase of synthesis. AI-assisted continuous microfluidic manufacturing has emerged as a promising strategy for addressing stability challenges in lipid nanoparticle production. By integrating real-time sensors with ML-based control systems, microfluidic platforms can dynamically monitor and adjust process parameters such as flow rate, temperature gradients, and mixing conditions to maintain optimal synthesis environments. These adaptive control strategies help ensure consistent nanoparticle formation and reduce batch-to-batch variability in CQAs such as particle size, polydispersity, and encapsulation efficiency [79,81].

In advanced implementations, RL or other AI-driven control algorithms can be used to interpret continuous sensor feedback and autonomously optimize operating conditions in real time. Such systems can rapidly adjust parameters, including thermal profiles or reaction conditions, to maintain the desired physicochemical state of lipid assemblies and prevent instability events that may compromise nanoparticle integrity. The ability to perform millisecond-scale adaptive control is particularly valuable in microscale reactors, where small fluctuations in temperature or flow can significantly influence lipid self-assembly and drug encapsulation efficiency [114,115].

The integration of AI into SLN synthesis has also increasingly focused on improving the long-term stability of lipid-based carriers [82]. In lipid-based nanocarriers, instability often arises from phase transitions within the lipid matrix, which can lead to structural rearrangement and subsequent drug expulsion. AI-assisted predictive frameworks allow researchers to anticipate these transitions by analyzing formulation variables and physicochemical characteristics, thereby enabling the proactive selection of lipid compositions and surfactant systems that stabilize the nanoparticle structure. This data-driven strategy facilitates rapid optimization of lipid–surfactant combinations to improve carrier stability and drug retention [116].

More recently, self-driving experimental platforms that integrate machine learning with automated experimentation have emerged in formulation science. These systems iteratively generate experimental data, update predictive models, and propose new candidate formulations with enhanced performance. By exploring large formulation spaces computationally before experimental validation, such platforms can accelerate the discovery of optimized lipid nanoparticle systems and improve the stability and delivery efficiency of encapsulated therapeutics [82,116].

A notable study illustrating these capabilities is the work of Osouli-Bostanabad et al. [79], which provided a comprehensive evaluation of microfluidic manufacturing strategies for lipid-based nanomedicines, including solid lipid nanoparticles (SLNs). It was demonstrated that continuous-flow microfluidic platforms, through precise control of FRR and TFR, can produce SLNs with tunable sizes ranging from 50 to 200 nm and consistently low polydispersity indices (PDI < 0.15). Critically, the study highlighted that the SLN size landscape is highly non-linear with respect to processing parameters, forming a complex response surface in the FRR-TFR space that is difficult to navigate using conventional trial-and-error approaches.

This work illustrates why AI-assisted methods are useful for SLN manufacturing. They can identify narrow operating windows in nonlinear FRR-TFR design spaces while reducing reliance on trial-and-error screening.

### 3.4. Particle-Size Control

Precise particle-size control is a critical requirement for effective drug delivery because in vivo pharmacokinetics and biodistribution are closely linked to hydrodynamic diameter. AI-integrated microfluidic systems can support flow-rate-driven tuning, often targeting nanoscale ranges relevant to tumor accumulation and cellular uptake. However, claims about an optimal 80–120 nm range or very low PDI thresholds should be framed as formulation- and indication-dependent rather than universal. Using BO, these platforms can efficiently identify combinations of TFR and FRR that approach desired particle-size and PDI targets [36].

**Figure 6:**
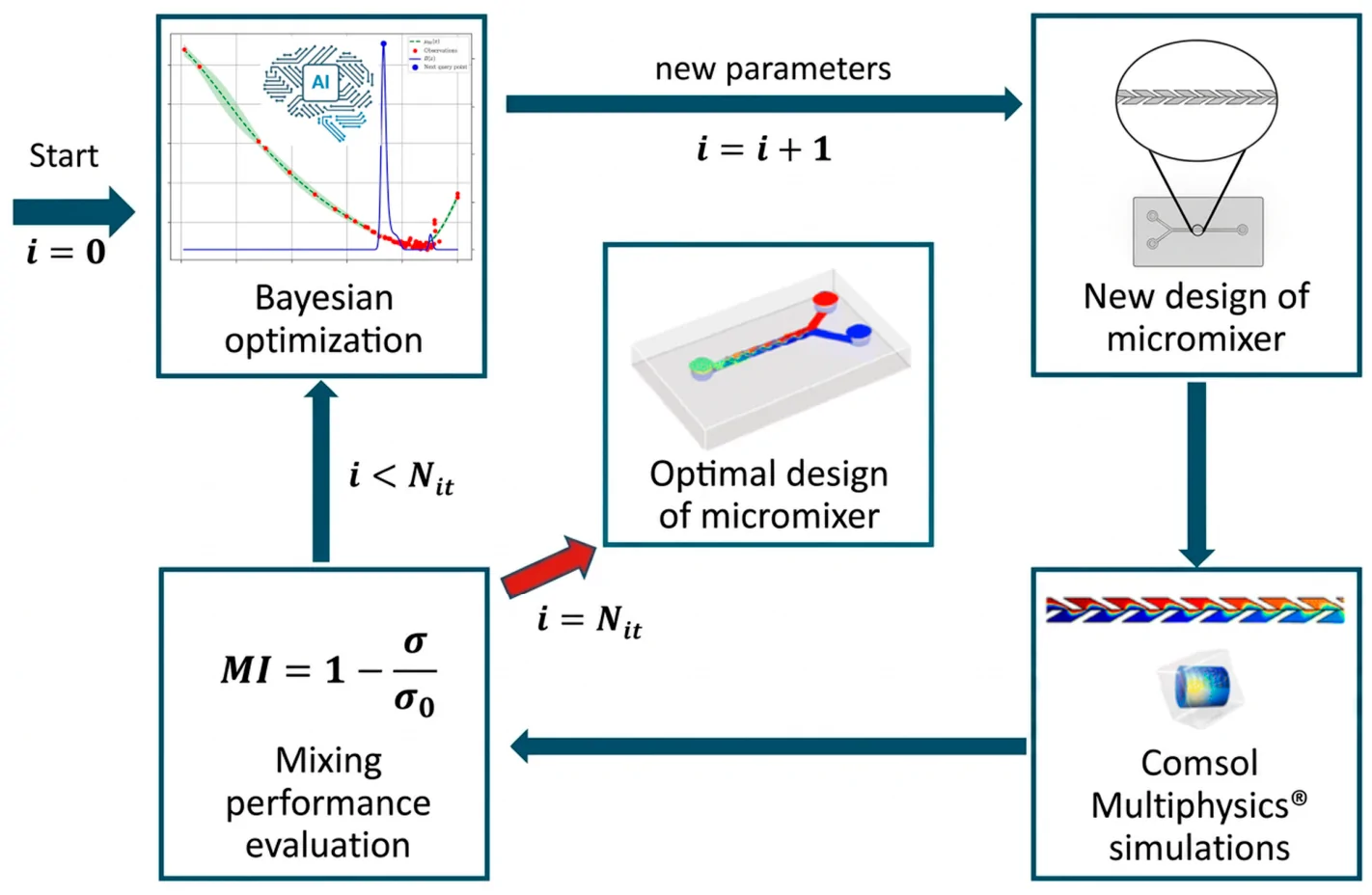
Schematic of the Bayesian Optimization (BO) workflow for iterative design and evaluation of micromixers in Comsol Multiphysics^®^ to achieve optimal design and mixing performance [36] (open-access CC-BY; no permission required).

For example, Kundacina et al. [36] demonstrated the application of BO in the automated design and tuning of microfluidic systems for nanoparticle production (Figure 6). Using a Gaussian Process surrogate model, the study showed that optimal conditions for producing nanoparticles within the desired size range could be identified in fewer than 15 experimental iterations, significantly faster than conventional search strategies, while also reducing reagent consumption. The incorporation of uncertainty quantification further enabled efficient exploration of the parameter space and robust identification of true optima despite experimental noise.

In adaptive manufacturing, in-line DLS or related sensors can detect particle-size deviations caused by reagent or environmental variation. AI controllers can then adjust flow parameters to keep products within predefined specifications, a capability that will be important if decentralized or point-of-care manufacturing becomes clinically feasible [39].

## 4. AI in Microfluidic Phenotypic Screening and Drug Testing

AI-enabled microfluidic screening extends conventional high-throughput screening by capturing richer single-cell and multicellular phenotypes. Instead of relying only on simplified bind/no-bind assays, these platforms can combine controlled microenvironments with DL-based image analysis to detect morphological, metabolic, and functional responses to drug candidates [117,118]. Microfluidic devices add miniaturization, automation, and parallelization, enabling concentration gradients and multiplexed cell-based assays with low reagent consumption [119]. Active learning can further reduce redundancy in screening by selecting the most informative compounds or dose combinations for subsequent testing, concentrating experimental effort where it is likely to be most valuable [52].

### 4.1. High-Content Screening (HCS) on Chips

Microfluidic HCS platforms integrate cell-culture chambers with imaging systems to monitor multiple experimental conditions using small sample volumes [120,121]. DL-based analysis can then process large image streams and detect drug-induced morphological or cellular responses with reduced manual annotation [122]. A key advantage of AI-driven HCS is sensitivity to subtle phenotypic signatures, including mitochondrial, cytoskeletal, or nuclear changes that may indicate early toxicity or therapeutic efficacy [122]. For nanomedicine, this combination is particularly useful because carrier performance often depends on cellular uptake, intracellular trafficking, and time-dependent biological response rather than a single endpoint measurement.

In the study by Yadav et al. on AI-driven HCS [121], the authors introduced a magnetofluidic on-chip platform that integrates capacitive sensor arrays with an ML classifier to predict combination drug efficacy. The system enables parallel testing of multiple drug combinations and concentrations within an array of microfluidic wells, while real-time impedance measurements provide quantitative, label-free readouts of cell viability. The trained ML model processes sensor data to generate two-dimensional drug–drug interaction heatmaps, classifying synergistic, additive, or antagonistic effects with over 90% accuracy compared with standard cytotoxicity assays. The platform can evaluate dozens of drug combinations per chip run while using sub-microliter reagent volumes per test, making it well suited for early-stage nanomedicine screening.

### 4.2. Organ-on-Chip (OoC) Models

Organ-on-a-chip (OoC) technology recreates selected physiological functions, such as epithelial barrier activity, cardiac contraction, or liver metabolism, within microfluidic devices that mimic aspects of the in vivo microenvironment [9,123]. When combined with AI, OoC platforms can analyze biosensor and imaging outputs to provide real-time indicators of tissue viability, barrier integrity, and organ-level response [124,125].

Crucially, high-content phenotypic data generated by OoC models can be directly fed back into AI-guided DDS design and formulation optimization, establishing a biologically driven closed-loop discovery platform. Rather than optimizing nanoparticles solely for physical attributes like particle size or encapsulation efficiency, the optimization loop is driven by biological performance [9,124,125]. For instance, in-line capacitive sensors in a liver-on-a-chip or a blood–brain barrier (BBB) model can measure trans-epithelial electrical resistance (TEER) in real time to monitor barrier integrity. Simultaneously, CNN-based image processing can classify tumor cell death or drug resistance states following nanocarrier perfusion. These biological outputs (e.g., maximizing cancer cell death while minimizing liver toxicity and maintaining BBB integrity) are converted into multi-objective fitness scores. Active learning or reinforcement learning algorithms process these scores to design the next generation of nanocarriers, iteratively adjusting lipid compositions or PEGylation densities. This direct integration ensures that the self-driving laboratory optimizes for functional therapeutic efficacy under biomimetic physiological flow, significantly improving preclinical translation.

**Figure 7:**
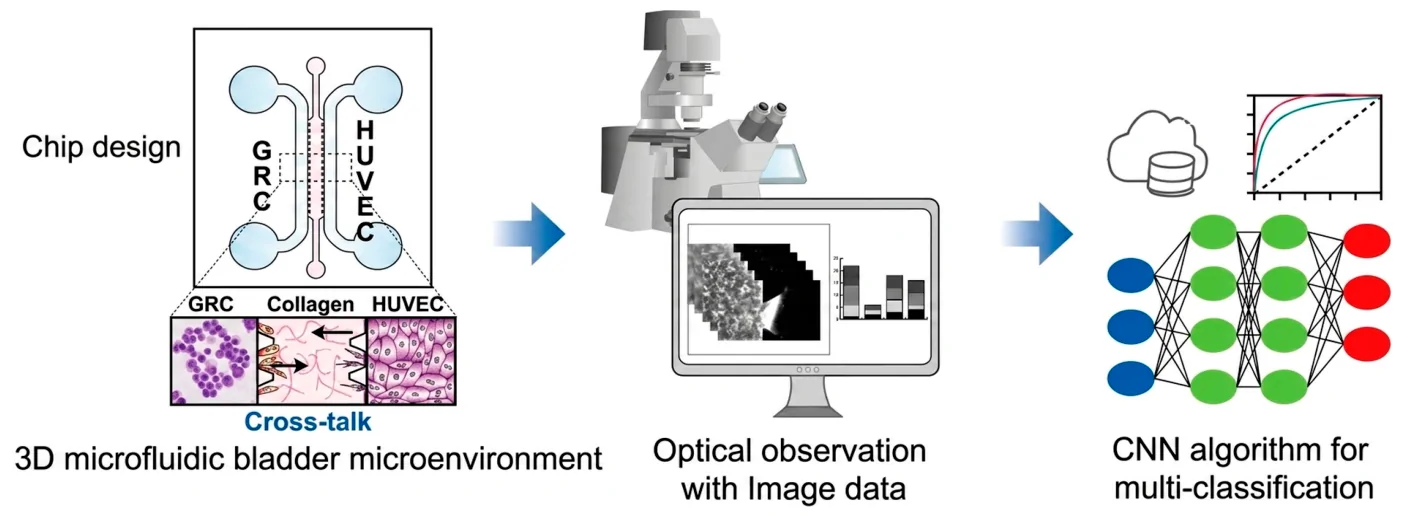
Schematic of the three-layer 3D microfluidic Organ-on-a-Chip (OoC) bladder cancer model coupled with a high-content imaging and CNN classification pipeline for drug resistance prediction [127] (open-access CC-BY; no permission required).

Multi-organ-on-chip systems extend this concept by linking chambers that simulate inter-organ interactions, enabling controlled studies of pharmacokinetics, metabolism, and toxicity that may better approximate human physiology than conventional cell culture [125,126]. In oncology research, three-dimensional (3D) microfluidic tumor models combined with DL methods are emerging as useful platforms for studying cancer progression and drug resistance. CNNs can analyze high-content imaging data from cancer-on-chip systems to classify morphological changes, migration patterns, and cell–microenvironment interactions associated with drug resistance. For example, Tak et al. [127] demonstrated an AI-assisted OoC platform using a three-layer, three-dimensional microfluidic bladder-cancer model comprising a vascular channel, a tumor matrix seeded with patient-derived cells, and a drug-perfusion channel. Following treatment with varying concentrations of cisplatin, high-content brightfield and fluorescence images of the tumor compartment were acquired and analyzed using a CNN trained to classify cells as sensitive, moderately resistant, or highly resistant based on morphological features (Figure 7).

The reported accuracy highlights the analytical value of combining OoC models with DL-based image interpretation, particularly for phenotypes that are difficult to distinguish using conventional viability assays alone. Patient-derived tumor-on-chip systems may ultimately help personalize therapy selection by testing tumor response in biomimetic microenvironments before clinical intervention, although further validation is required before routine clinical use [126,127].

## 5. Regulatory and Practical Challenges in AI-Integrated Microfluidics for Drug Delivery

The emergence of AI-integrated hardware–software systems in pharmaceutical manufacturing requires regulatory frameworks that can evaluate adaptive, software-mediated manufacturing processes. Traditional regulatory pathways were generally designed for static manufacturing processes and conventional medical devices, and may be less suited to systems in which AI algorithms update operational parameters during production. Because such systems combine autonomous software agents with physical manufacturing platforms, they may not fit neatly into existing regulatory categories. Regulatory bodies therefore need evaluation strategies that assess safety, efficacy, robustness, and lifecycle stability of dynamic AI-enabled manufacturing systems [39,40].

Regulatory evaluation must therefore address device validation, algorithmic performance, training-data provenance, sensor reliability, and safeguards that prevent autonomous systems from operating outside acceptable process boundaries [128,129]. For adaptive systems, validation should focus not only on a fixed operating point but also on the defined design space within which the AI model is permitted to act, together with documentation of software architecture, training data, in-line monitoring, and lifecycle change control [39,40].

### 5.1. Current Challenges and Bottlenecks

#### 5.1.1. Reproducibility and Data Scarcity

A persistent challenge in AI-driven pharmaceutical manufacturing is that automation does not eliminate all sources of experimental variability. Even in highly automated continuous manufacturing systems, variability can arise from raw material heterogeneity, equipment disturbances, and environmental fluctuations, requiring robust monitoring and adaptive control strategies [130,131,132]. Despite the high precision of microfluidic platforms, reproducibility can still be affected by impurities in raw materials, variations in precursor preparation, and batch-to-batch differences in chemical reagents. Even sophisticated AI systems cannot compensate for inconsistencies in parameters such as polymer molecular weight distributions or lipid oxidation levels if these variables are not captured by the sensor infrastructure of the system.

In addition, the field faces a substantial data scarcity problem. Many ML models used in pharmaceutical development are trained on small or proprietary datasets generated within individual laboratories or companies, limiting the generalizability of these models across different experimental setups and manufacturing environments. The lack of standardized, publicly available datasets for nanoparticle synthesis and microfluidic processes significantly restricts the scalability and reproducibility of AI-driven approaches [133,134].

#### 5.1.2. Scalability, Hardware Reliability, and Material Compatibility

Another major bottleneck is the transition from laboratory-scale microfluidic systems to industrial-scale pharmaceutical manufacturing. Microfluidic devices are highly efficient for producing nanoliter- or picoliter-scale formulations with exceptional control over particle size, morphology, and composition [135]. However, translating these platforms to produce clinically relevant volumes, such as liters of vaccine formulations, remains a significant engineering challenge. To address this limitation, strategies such as numbering-up or scale-out are commonly employed, in which large arrays (hundreds or thousands) of identical microfluidic reactors operate in parallel to increase total production capacity while preserving the precise flow characteristics of microscale reactors [23,135,136].

At the same time, stacking and parallelizing devices in 3D architectures introduces severe engineering and physical bottlenecks, including (i) uniform fluid distribution: ensuring that fluid flow rates are perfectly identical across thousands of parallel channels is highly challenging, as minor manufacturing tolerances or local clogs can cause fluidic maldistribution and ruin nanoparticle homogeneity; (ii) cumulative pressure drop: as layers are added, the overall flow resistance increases, leading to massive cumulative pressure drops that can exceed pump limits or rupture seals; and (iii) bonding reliability: maintaining leak-free, high-pressure bonding between multiple stacked layers of elastomer or thermoplastic is difficult, particularly under prolonged continuous operation.

Furthermore, substrate material compatibility represents a major bottleneck for drug delivery synthesis. Traditional laboratory devices are fabricated from polydimethylsiloxane (PDMS) due to its ease of rapid prototyping [135]. However, PDMS exhibits poor compatibility with organic solvents such as dimethyl carbonate (DMC), dichloromethane (DCM), or ethyl acetate, which are standard in polymeric and lipid nanoparticle synthesis. These solvents cause severe PDMS swelling, which deforms channel morphology, alters flow-focusing dimensions, and leads to device failure after brief operations. To transition to mass production, the field must shift toward thermoplastic substrates (such as cyclic olefin copolymer [COC], polymethyl methacrylate [PMMA]) or glass. These materials offer superior chemical resistance, rigid channel dimensions under high pressure, and established manufacturing pathways (like injection molding), making them highly suitable for industrial-scale, AI-monitored manufacturing.

While numbering-up preserves the advantages of microfluidic precision, it also introduces significant engineering challenges. Parallelized microfluidic systems are susceptible to clogging, leakage, channel fouling, and pressure fluctuations during prolonged operations. Ensuring uniformity and reliability across large arrays of devices requires sophisticated monitoring systems capable of detecting faults within individual channels without compromising the integrity of the entire production batch. AI-driven monitoring and predictive maintenance strategies have been proposed to address these issues, but they introduce additional computational and infrastructural complexity [135,136].

#### 5.1.3. Explainability, Clinical Trust and Translational Stage

The black-box nature of many DL models remains a major barrier to regulatory acceptance and clinical trust. In pharmaceutical development, predictions generated by AI systems must be interpretable enough for scientists, clinicians, and regulators to understand the mechanistic basis behind model recommendations.

To foster this trust, we must explicitly clarify the translational stage of different AI and nanomedicine claims, distinguishing verified clinical evidence from preclinical laboratory findings and future projections:

(i) Clinical Evidence: Clinical proof is currently limited to the delivery vehicles themselves. Formulations like FDA-approved mRNA-LNP vaccines (Comirnaty and Spikevax) and patisiran (Onpattro) have robust clinical efficacy, though their industrial manufacture did not natively utilize real-time AI optimization.
(ii) Laboratory and Preclinical Studies: The vast majority of AI-microfluidic integrations—such as closed-loop LNP optimization, CNN-based droplet sorting, and on-chip toxicity prediction—are strictly at the preclinical, in vitro stage. These systems are highly effective in laboratory settings but have not yet entered clinical trials.
(iii) Future Projections: Fully autonomous self-driving clinics, decentralized point-of-care synthesis of patient-specific therapies, and complete digital-twin-driven manufacturing systems are long-term projections. These concepts require significant engineering standardisation, clinical validation, and regulatory approval before clinical implementation.

XAI approaches, such as SHAP, feature attribution maps, and model-agnostic interpretability frameworks, are increasingly used to address this challenge [80,102]. These tools enable researchers to identify which input variables most strongly influence model predictions, thereby improving transparency and supporting regulatory evaluation. However, achieving a balance between high-performance deep learning models and interpretable models suitable for regulatory review remains an ongoing challenge in AI-assisted pharmaceutical research [137,138,139].

### 5.2. FDA Framework for AI in Drug Development

To address regulatory challenges posed by AI-driven systems, the U.S. Food and Drug Administration (FDA) has proposed a risk-based credibility assessment framework for AI models used in drug development and regulatory decision-making [39]. This framework is intended to ensure that AI systems meet appropriate standards for transparency, reliability, and reproducibility before integration into pharmaceutical workflows. The process begins by defining the Question of Interest and Context of Use (COU), which specify the operational scope of the AI model and the decision it is intended to support. The risk level is then evaluated according to the potential consequences of incorrect predictions for drug quality or patient safety. Developers subsequently prepare a Credibility Assessment Plan that describes data management, model training, hyperparameter optimization, and validation protocols. Regulators can then evaluate whether the model performs consistently across relevant datasets, manufacturing platforms, and experimental conditions.

This structured evaluation process provides a pathway for integrating AI into advanced pharmaceutical manufacturing processes, including the production of lipid nanoparticles, polymeric nanocarriers, and other complex drug delivery systems. By aligning AI-assisted manufacturing systems with current Good Manufacturing Practices (cGMPs) and regulatory expectations, the framework supports the development of closed-loop pharmaceutical production platforms that are both efficient and compliant with regulatory standards [39,40].

## 6. Future Perspectives

Future AI–microfluidic DDS platforms are likely to develop along three connected directions: edge-enabled process control, digital twins for predictive maintenance and design, and decentralized manufacturing for selected therapeutic contexts [140,141]. Cloud-connected diagnostic and manufacturing systems may eventually link patient biomarker data with formulation or dosing decisions, while synthetic-biology approaches could add living, programmable delivery systems that respond to physiological signals [142,143,144].

### 6.1. Personalized Pharmacy, Point-of-Care Synthesis, and Machine-Vision Monitoring

Personalized pharmacy envisions drug formulations produced closer to the point of care rather than exclusively in centralized facilities. AI-controlled microreactors in hospitals or clinics could, in principle, produce customized dosages or formulations from real-time biomarker feedback, which may be especially relevant for unstable biologics and mRNA-based therapeutics [110,136].

To make high-throughput, continuous point-of-care manufacturing practically viable, platforms must integrate machine-vision-based online droplet/particle quality monitoring and closed-loop regulation. Currently, most particle-size measurements rely on offline microscopic imaging or batch DLS post-processing, which cannot catch real-time drift during continuous operation. Incorporating high-speed in-line cameras coupled with real-time CNNs provides a powerful technical solution.

These deep learning algorithms can process video feeds of microchannels at millisecond scales, performing automated online particle size recognition, morphology classification, and defect detection (such as identifying droplet coalescence, polydispersity spikes, or minor channel clogging). Upon detecting a defect, the AI controller immediately calculates and executes necessary flow adjustments (via closed-loop feedback loops connected to electronic syringe pumps or thermal controllers) to restore product uniformity. This transition from offline inspection to active, machine-vision-guided online quality control represents a critical step for the clinical deployment of autonomous manufacturing platforms.

Realizing this vision will require plug-and-play cartridges, validated quality-control modules, robust operator safeguards, and integration with digital health records. AI would serve as the supervisory layer for monitoring, release testing, and process adjustment [39,142,145].

### 6.2. Sustainability and Green Pharmaceutics

With increasing global pressure to reduce the environmental footprint of pharmaceutical manufacturing, green pharmaceutics has emerged as a key research priority. AI-optimized microreactors offer a promising pathway toward sustainable production by significantly reducing solvent consumption, reaction waste, and energy requirements. In contrast to conventional batch synthesis, which often generates substantial chemical by-products, AI-driven continuous-flow systems can dynamically optimize reaction parameters and stoichiometry to maximize precursor conversion and minimize waste streams [146,147].

Additionally, ML algorithms are increasingly used to identify environmentally benign solvents and bio-derived materials for nanoparticle synthesis and drug formulation. By screening large chemical spaces and predicting reaction efficiencies, these models can identify low-carbon synthesis pathways that maintain therapeutic efficacy while minimizing environmental impact. The integration of microfluidic precision with AI-based sustainability optimization therefore enables pharmaceutical manufacturing processes that are both clinically effective and environmentally responsible [148].

### 6.3. Human-in-the-Loop and Future Frontiers

Despite rapid progress in automation, the future of AI-enabled drug discovery is likely to rely on Human-in-the-Loop frameworks that combine machine intelligence with expert scientific oversight. In this collaborative paradigm, AI systems function as decision-support tools that analyze large datasets, identify hidden experimental patterns, and propose novel experimental strategies that might otherwise be overlooked by human researchers. Scientists, in turn, provide contextual interpretation, hypothesis generation, and ethical oversight.

This collaboration is particularly important for emerging research frontiers such as self-driving laboratories, which integrate robotics, ML, and automated experimentation to autonomously design and perform chemical or biological experiments [90]. Future systems may also incorporate self-healing microfluidic architectures, adaptive biosensors, and programmable gene circuits capable of detecting and responding to disease signals within the body. As these technologies mature, biomedical engineers will increasingly focus on designing the objective functions, safety constraints, and ethical frameworks that guide autonomous experimental systems, while improving the awareness of the importance of collaboration and knowledge sharing, to ensure that technological progress remains aligned with clinical and societal priorities [144,149].

### 6.4. Conclusions

The integration of AI and microfluidics has established a new paradigm in pharmaceutical engineering and drug delivery research. In addition, advances in additive manufacturing enable the fabrication of three-dimensional microfluidic architectures that are difficult or impossible to produce using conventional lithographic techniques. These complex geometries can be optimized using AI-assisted simulations and physics-informed neural networks, allowing precise control over droplet formation, mixing efficiency, and reaction kinetics within microfluidic reactors.

Ultimately, the convergence of AI with microfluidic technologies offers a powerful framework for accelerating drug discovery and improving therapeutic precision. By combining microscale fluid control with predictive modeling, researchers can automate high-content screening, optimize drug formulations, and develop sophisticated organ-on-chip platforms for toxicity and efficacy testing. Although challenges remain, including data privacy concerns, algorithmic bias, and evolving regulatory frameworks, the emergence of self-driving laboratories and AI-assisted manufacturing systems promises to reduce experimental variability and accelerate the translation of personalized therapies into clinical practice. As these technologies mature, they are poised to redefine the future of healthcare, enabling increasingly precise, adaptive, and patient-specific therapeutic interventions, and advancing personalized medicine.

## Figures and Tables

**Table 1:** Critical Quality Attributes (CQAs), Microfluidic Parameters, and AI Optimization Strategies.

Critical Quality Attribute (CQA)	Key Microfluidic/Synthesis Parameters	Primary AI Modeling & Optimization Strategy	Literature Exemplars/Implementations	Development Stage
Particle Size (Hydrodynamic Diameter)	Flow Rate Ratio (FRR), Total Flow Rate (TFR), Lipid/Polymer Concentration	Bayesian Optimization (BO) with Gaussian Process (GP) surrogate; Supervised ANNs	Kundacina et al. [36] optimized size in <15 runs; Rebollo et al. [78] achieved R^2^ > 0.97 using ANNs	Experimentally Demonstrated
Polydispersity Index (PDI)	FRR, TFR, Microfluidic channel geometry (MHF vs. SHM), Solvent composition	Gaussian Process Regression, Random Forest, Deep Neural Networks	Osouli-Bostanabad et al. [79] mapped non-linear PDI surfaces; Buttitta et al. [69] optimized liposomes	Experimentally Demonstrated
Encapsulation Efficiency (EE)	Polymer/lipid concentration, Drug-to-lipid ratio, Organic-to-aqueous phase ratio	Supervised regression models (Random Forest, SVR, Gradient Boosting)	Hanari et al. [80] achieved R^2^ = 0.96 for PLGA nanocarrier EE utilizing Random Forest	Experimentally Demonstrated
Drug Loading (DL)	Initial drug concentration, polymer chemistry (LA/GA ratio, MW), solvent type	Supervised learning, SHAP-based feature importance, Random Forest	Hanari et al. [80] achieved R^2^ = 0.93; identified polymer MW and LA/GA ratio as critical	Experimentally Demonstrated
Long-term Stability & Gelation	Storage temperature, lipid composition, surfactant concentration, thermal gradients	Reinforcement Learning (RL) for active thermal control; Deep RL	Sato et al. [81] and Dorsey et al. [82] predicted phase transitions and gelation during SLN cooling	Predictive/In Silico

## Data Availability

No new data were created or analyzed in this study. Data sharing is not applicable to this article.
